# OH, YOU JUST GOT ARTHRITIS: OLDER AFRICAN AMERICANS LACK FAMILIAL, SOCIAL, AND PROVIDER SUPPORT FOR PAIN MANAGEMENT

**DOI:** 10.1093/geroni/igz038.272

**Published:** 2019-11-08

**Authors:** Staja Booker, Keela Herr, Toni Tripp-Reimer

**Affiliations:** 1 The University of Florida, Gainesville, Florida, United States; 2 The University of Iowa College of Nursing, Iowa City, Iowa, United States

## Abstract

Self-management support from family, friends, and providers is a crucial element in controlling osteoarthritis pain. 110 African-Americans (50-94 years) were surveyed regarding social and provider self-management support, and 18 of the African-American participants were also individually interviewed. This mixed-methods analysis unveiled that 77% were not receiving familial/social or provider support, and a conventional qualitative content analysis confirmed the lack of expected support for self-management with sentiments such as “I’m doin’ this all on my own.” Nonetheless, older African-Americans respected providers’ professional opinion, and 82% believed that treatment from a provider would be helpful. They desired more education and treatment options because they “need somebody to help with these joints and muscles”. However, participants were forced to learn how to care for osteoarthritis pain: “I was taking pain medication, but when I went to the doctor last time he told me to stop… Told me to deal with it [pain].”

